# Research progress on TWEAK/Fn14 signaling in chronic wound healing

**DOI:** 10.3389/fsurg.2026.1769996

**Published:** 2026-04-29

**Authors:** Bo Liu, Weimin Wu, Weigang Hu, Huarong Zheng, Tianyao Lan

**Affiliations:** 1 Department of Burn and Plastic Surgery, The First College of Clinical Medical Science, China Three Gorges University, Yichang, Hubei Province, China; 2 Department of Burn and Plastic Surgery, Yichang Central People’s Hospital, Yichang, China

**Keywords:** chronic wounds, Fn14, therapeutic target, TWEAK, wound healing

## Abstract

Chronic wounds are characterized by persistent inflammation and altered microenvironments, exhibiting prolonged healing and difficult repair, presenting significant therapeutic challenges. Tumor necrosis factor-like weak inducer of apoptosis (TWEAK), by binding to its receptor fibroblast growth factor-inducible 14 (Fn14), participates in cellular regulation, wound repair, and inflammatory response processes, playing an important role in chronic wound healing. This review summarizes the pathophysiological mechanisms of chronic wounds, the mechanisms of action of the TWEAK/Fn14 signaling pathway in chronic wound healing, and the latest research progress on its potential as a therapeutic target.

## 1 Introduction

Clinically, skin wounds that fail to heal after 4 weeks or wounds that persistently recur without apparent healing tendency are defined as chronic refractory wounds (CRW) (1). According to statistics, approximately 1%–2% of the global population is susceptible to injury during their lifetime. With advancing age, the incidence of chronic wounds increases, with a prevalence of 3.6% in individuals over 60 years and up to 6% in those over 80 years (2). These wounds may be caused by diabetes, varicose veins, arterial disease, pressure injuries, and radiation therapy injuries (3), with the proportion of diabetes-induced wounds gradually increasing, significantly impacting patients’ quality of life, healthcare systems, and socioeconomic burden.

Tumor necrosis factor-like weak inducer of apoptosis (TWEAK) is a member of the tumor necrosis factor (TNF) superfamily, and fibroblast growth factor-inducible 14 (Fn14) is the only known receptor for TWEAK. Current research has demonstrated that TWEAK/Fn14 participates in and regulates cell proliferation, migration, apoptosis, neovascularization, and inflammatory responses. In normal cells and tissues, TWEAK/Fn14 expression is typically low; however, under conditions of injury, oxidative stress, and inflammation, TWEAK/Fn14 expression is upregulated (4). In recent years, accumulating evidence indicates that the TWEAK/Fn14 signaling pathway plays a crucial role in tissue repair and regeneration processes (5, 6). This review examines chronic wound healing and the mechanisms of TWEAK/Fn14 in chronic wounds.

## 2 Major processes of normal wound healing

Wound healing is an extremely complex process, involving sequential and overlapping stages of hemostasis, inflammation, proliferation, and remodeling, with each stage involving multiple cells and cytokines. Immediately after wound formation, hemostasis begins, with vasoconstriction and the coagulation cascade initiated first, while platelets rapidly adhere and activate, releasing various cytokines to accelerate wound healing (7). Subsequently, the inflammatory phase begins. In the early stage, neutrophils migrate in large numbers to the wound through chemotaxis, releasing reactive oxygen species (ROS), proteolytic enzymes, and lysosomal enzymes to eliminate pathogens and cellular debris (7, 8). In the later stage, monocytes migrate within the wound and differentiate into macrophages, continuing to phagocytose bacteria and cellular debris while secreting large amounts of pro-inflammatory and anti-inflammatory cytokines (such as IL-1, TNF-α, TGF-*β*), regulating the inflammatory microenvironment and promoting vascularization and fibroblast proliferation during the proliferative phase through secretion of vascular endothelial growth factor (VEGF) and fibroblast growth factor (FGF), thereby promoting wound healing (7, 9). The main processes of the proliferative phase are re-epithelialization, angiogenesis, and granulation tissue formation. Keratinocytes and epithelial cells migrate and proliferate, promoting wound healing, while vascular endothelial cells are activated and proliferate in response to multiple growth factors, forming new blood vessels. During this stage, granulation tissue forms in the wound area, filling from the wound base upward and gradually covering the wound (10). The remodeling phase is characterized by the replacement of type III collagen produced in the extracellular matrix (ECM) with type I collagen, which has higher tensile strength but requires longer deposition time. Accompanied by a decrease in the number of blood vessels, a mature avascular environment is formed. Simultaneously, Mechanical tension and cytokines (such as TGF-*β*) drive fibroblast differentiation into myofibroblasts, which express *α*-smooth muscle actin(*α*-SMA) and contract the wound (11).

## 3 TWEAK/Fn14 structure and related signaling pathways in chronic wounds

### 3.1 Overview of the TWEAK/Fn14 signaling pathway

**Figure 1:**
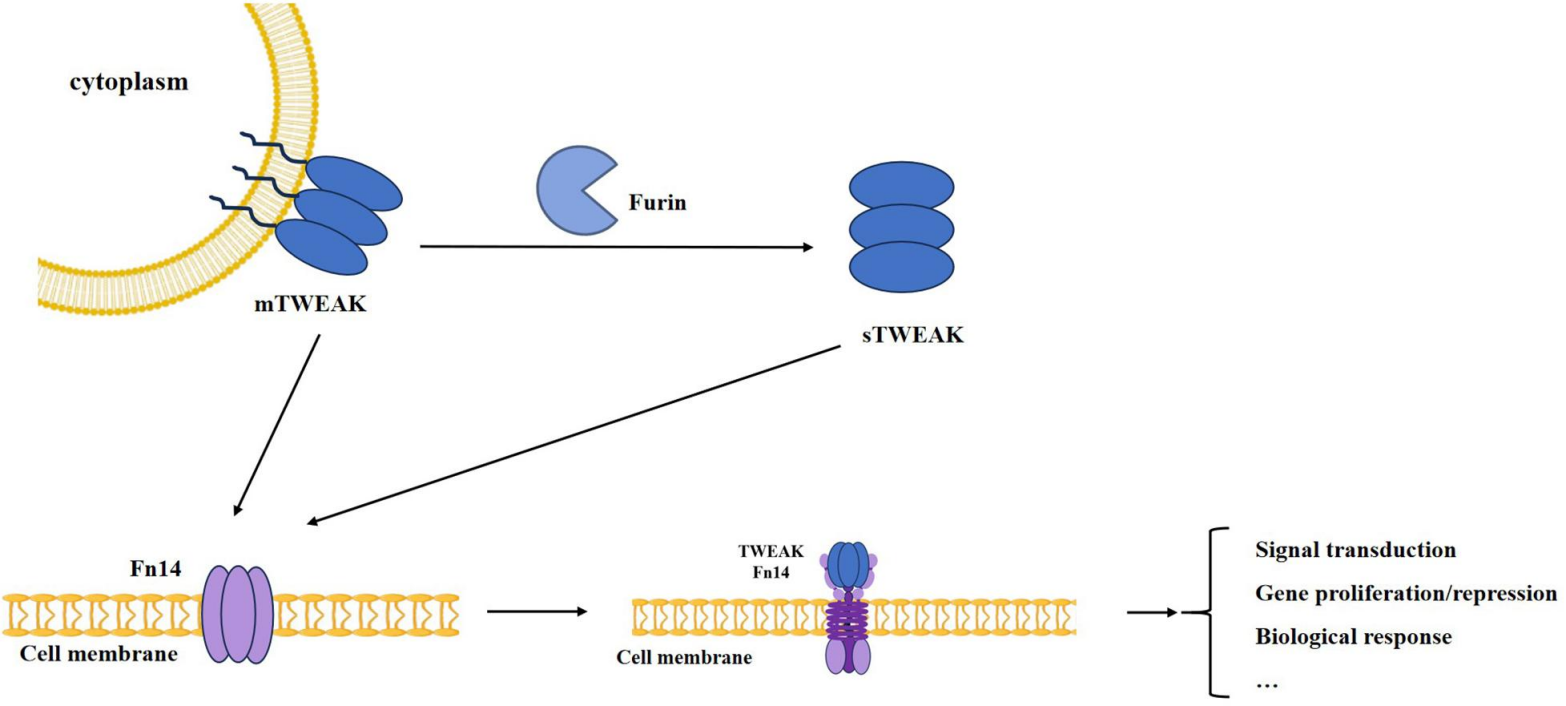
TWEAK/Fn14 signaling pathway.

In 1997, TWEAK was first identified as a novel, highly conserved pro-apoptotic TNF-like protein in HT-29 human colon cancer cells treated with interferon gamma (IFNg) (12). TWEAK, as a type II transmembrane protein, contains a typical TNF homology domain (THD) in its extracellular segment. This domain comprises 249 amino acids and forms a stable trimeric structure through *β*-sheet folding(13). It includes an N-terminal intracellular domain containing potential protein kinase C phosphorylation sites, a transmembrane domain responsible for membrane anchorage, and a C-terminal extracellular domain containing a stalk region and a typical TNF homology region (14, 15). TWEAK exists in two forms: the membrane-bound form (mTWEAK) composed of 246 amino acids, and the soluble form (sTWEAK) formed after processing by Furin, which consists of 156 amino acids and is released into the circulation. Both forms can bind to Fn14 and trigger intracellular signal transduction (Figure 1). Besides Fn14, CD163 targets sTWEAK and mediates its degradation (13, 16–18). Fn14 is a type I transmembrane protein initially synthesized with 129 amino acids. Its structure includes an extracellular region containing a cysteine-rich domain (CRD), a transmembrane region that anchors the protein in the cell membrane, and an intracellular region containing a TRAF (TNF receptor-associated factor) binding motif.

**Figure 2:**
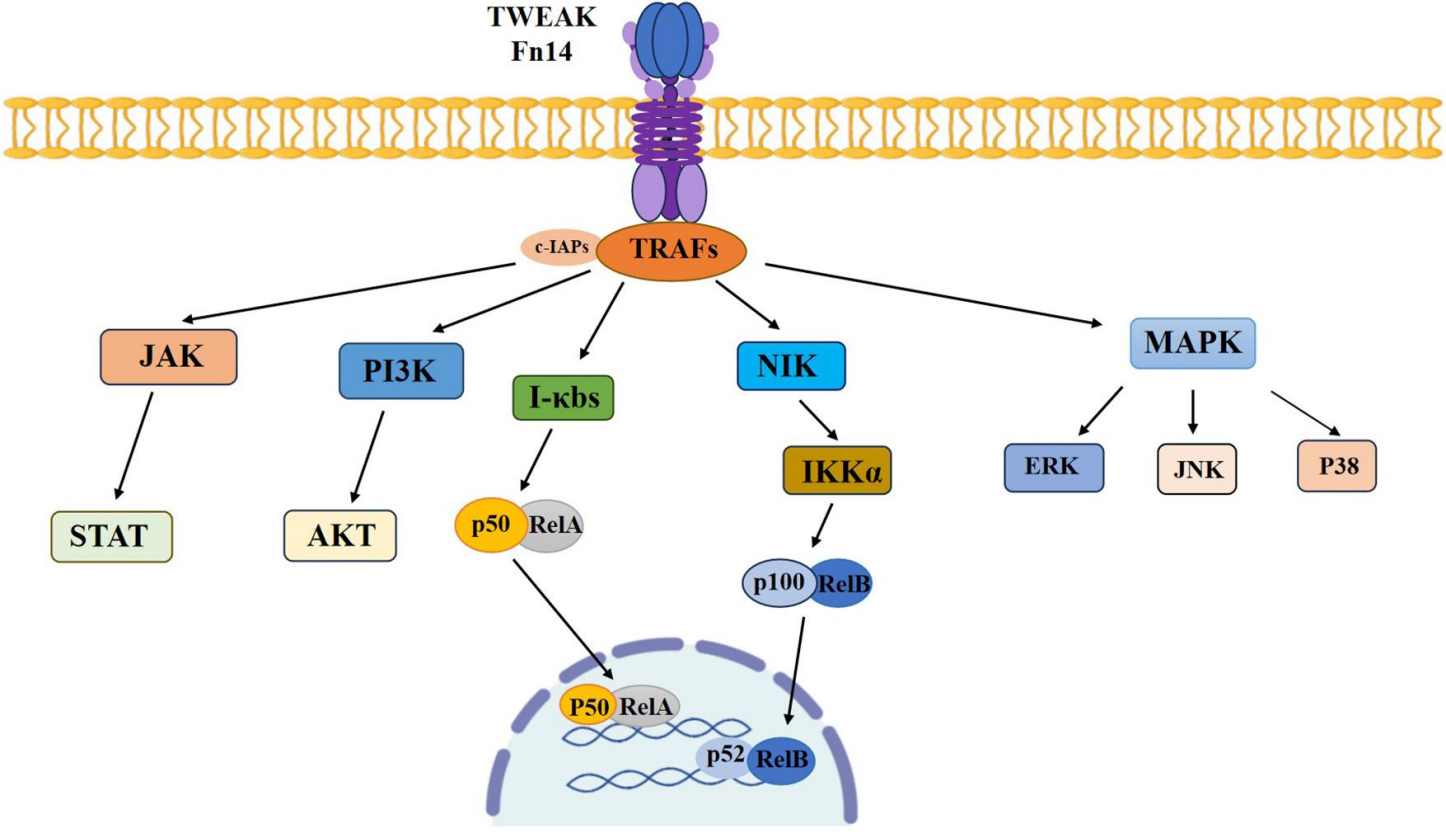
Signaling pathway activated by TWEAK/Fn14.

The TWEAK and Fn14 signaling pathways constitute a dynamic molecular cascade reaction, involving various biological processes ranging from tissue homeostasis to inflammation and cancer progression. TWEAK binding induces the trimerization of the Fn14 receptor, recruits TNF receptor-associated factors (TRAFs) and cell apoptosis inhibitory proteins (c-IAPs), activating multiple intracellular signaling cascades, including classical and non-classical NF-*κ*B, MAPK, PI3K/Akt and JAK/STAT, which are involved in the regulation of inflammatory components, ultimately leading to the regulation of cell survival and death, proliferation, differentiation, migration, invasion and angiogenesis (13, 16). The activation of the NF-*κ*B pathway is associated with the known functions of most TWEAK and the expression of pro-inflammatory cytokines, chemokines and cell adhesion molecules induced by TWEAK, controlling the expression of over 400 genes (19, 20). The classical NF-*κ*B pathway mainly involves the phosphorylation of NF-*κ*B inhibitory proteins (I*κ*B) and the nuclear translocation of the P50:RelA dimer, while the non-classical NF-*κ*B pathway includes the activation of kinase NIK, promoting the phosphorylation of downstream IKK*α* to produce p100, which ultimately induces the degradation of p100 into p52 and translocation to the nucleus to regulate transcription (21). Additionally, TWEAK/Fn14 can also induce the phosphorylation of the downstream MAPK pathway, mainly consisting of three main branches: extracellular regulated protein kinases (ERK), c-Jun N-terminal kinase (JNK), and p38 protein kinase (22), playing an important regulatory role in inflammatory responses, cell proliferation, and stress responses (23) (Figure 2).

### 3.2 Chronic wounds are associated with the TWEAK/Fn14 signaling pathway

Unlike normal wounds, chronic wounds are characterized by prolonged stagnation in the inflammatory stage due to multiple pathological factors, preventing progression to proliferation and remodeling stages, manifesting as exudation, recurrent infection, and tissue necrosis (24). Chronic wound healing is characterized by prolonged presence of myeloid cell populations such as macrophages, neutrophils, and monocytes during the late inflammatory phase. The wound remains enriched with M1 pro-inflammatory macrophages and neutrophils for extended periods, releasing large amounts of ROS and matrix metalloproteinases (MMPs) that continuously destroy tissue, accompanied by excessive expression of pro-inflammatory factors such as TNF-α, IL-1β, and IL-6, while the proportion of anti-inflammatory M2 macrophages decreases, impairing immune clearance and repair function. Simultaneously, the wound is occupied by biofilms, persistently activating pattern recognition receptors such as TLR and NLR, maintaining the inflammatory cycle (24). Additionally, senescent cells cannot be cleared promptly from the wound, secreting senescence-associated secretory phenotype (SASP) factors, exacerbating local inflammation and inhibiting fibroblast and keratinocyte function, prolonging wound healing (25). Keratinocytes play a crucial role in promoting wound healing. TWEAK and TNF-α work together to induce keratinocytes to express pro-inflammatory factors and chemokines such as IL-6, thereby activating normal T cells to induce inflammatory responses, regulating the expression and secretion of cytokines and granulocyte-macrophage colony-stimulating factor (26), and enhancing the synthesis of survivin and apoptosis inhibitor 2 (cIAP2), promoting the proliferation of keratinocytes (27). TWEAK can upregulate fibroblast growth factor-2, promoting the proliferation of dermal fibroblasts and collagen synthesis. Fibroblasts can secrete keratinocyte growth factor, stimulating the proliferation and migration of keratinocytes, thereby promoting the re-epithelialization of damaged tissues (28). At the same time, TWEAK can promote the production of various cytokines by macrophages to infiltrate the damaged tissue. After binding to the Fn14 on the intrinsic cells of the tissue, it promotes the migration, proliferation or differentiation of the intrinsic cells of the tissue, and also promotes the production of chemokines, including IL-6, IL-8, MCP-1, RANTES, IP-10, MMPs, etc., and recruits inflammatory cells including macrophages to the wound (6), accelerating the resolution of inflammation. Hypoxia is another characteristic of chronic wounds, accompanied by dysregulation of the HIF-1*α* pathway, insufficient expression or limited function of downstream pro-angiogenic factors such as VEGF and FGF, and elevated levels of anti-angiogenic factors (such as PEDF), resulting in sparse neocapillaries and microcirculatory dysfunction (29). TWEAK can upregulate the expression of vascular endothelial growth factor A, promoting increased vascular permeability, endothelial cell migration, proliferation and angiogenesis (30). Simultaneously, the activity of multiple matrix metalloproteinases is significantly increased, causing ECM degradation imbalance, affecting granulation tissue formation and tissue repair, and delaying or even blocking the healing process (31). The TGF-*β* signal increases the expression of Fn14 and the synthesis of extracellular matrix in dermal fibroblasts by activating the mediator Smad4 protein (32). Overall, these mechanisms often interact, forming a vicious cycle that makes the wound healing process difficult to proceed.

### 3.3 Related signaling pathways in chronic wounds

The phosphatidylinositol 3-kinase (PI3 K)/protein kinase B (AKT), extracellular factor/*β*-catenin, transforming growth factor-*β* (TGF-*β*), nuclear factor erythroid 2-related factor 2 (Nrf2), Notch, and hypoxia-inducible factor 1 (HIF-1) signaling pathways play key roles in wound healing by regulating the inflammatory, proliferative, and remodeling stages (33). The PI3K/AKT signaling pathway enhances epithelial-mesenchymal transition, cell proliferation, and angiogenesis while reducing inflammation after tissue injury (34, 35). When this pathway is inhibited, wound healing is impaired (35). The extracellular factor/*β*-catenin pathway activates stem cells, induces dermal and epidermal cell proliferation, and promotes angiogenesis by activating transcription of Wnt target genes, helping wound contraction and reducing fibrotic scar formation (36). The TGF-*β* pathway promotes granulation tissue formation and epidermal cell migration to cover wounds by regulating fibroblast and keratinocyte proliferation and migration after tissue injury, while also having scar-reducing effects (37). The role of Nrf2 in wound healing is to promote epithelial proliferation and migration while reducing oxidative stress and cell atrophy (38). The Notch signaling pathway regulates multiple cell types and processes, is crucial for proliferation in keratinocytes and endothelial cells, and plays a key role in recruiting M2 macrophages (33). HIF-1 signaling is activated in response totissue hypoxia, functioning to regulate angiogenesis and inflammation, promoting chronic wound healing (33). In addition, NF-*κ*B (nuclear factor-*κ*B) (39), Hippo/YAP (40, 41), Ras/Raf/MEK/ERK (42, 43), and JAK/STAT (43) signaling pathways also participate in chronic wound healing processes. This review summarizes the molecular mechanisms describing chronic wound healing (Table 1).

**Table 1:** Related signaling pathways in chronic wounds.

Signaling pathway	Biological effects
PI3K/AKT	Enhances epithelial-mesenchymal transition, cell proliferation and angiogenesis, reduces inflammation.
Extracellular factor/*β*- catenin	Induces dermal and epidermal cell proliferation and promotes angiogenesis, helps wound contraction and reduces fibrotic scar formation.
TGF-β	Regulates fibroblast and keratinocyte proliferation and migration, promotes granulation tissue formation, enables epidermal cell migration to cover wounds, reduces scarring.
Nrf2	Promotes epithelial proliferation and migration, reduces oxidative stress and cell atrophy
Notch	Promotes keratinocyte and endothelial cell proliferation, while recruiting M2 macrophages
HIF-1	Regulates angiogenesis and inflammation, promotes chronic wound healing
NF-*κ*B	Prolongs inflammation, reduces angiogenesis, reduces proliferation
Hippo/YAP	Regulates macrophage M2 polarization, promotes wound healing and angiogenesis
Ras/Raf/MEK/ERK (MAPK)	Regulates tissue homeostasis and development, inflammation, apoptosis, cell proliferation, differentiation, cytokine production and cell survival
JAK/STAT	Coordinates cellular responses to multiple cytokines and growth factors

### 3.4 TWEAK/Fn14 signaling is associated with other signals in chronic wounds

Signal transduction in the process of chronic wound healing is a complex process. These signaling pathways regulate biological processes such as cell proliferation, differentiation, migration, apoptosis, and inflammatory response, and jointly affect the progress of chronic wound healing. Currently, the mechanisms related to the TWEAK/Fn14 signaling pathway are still under continuous research. Epidermal growth factor receptor (EGFR) is a key growth factor receptor in the wound healing process, regulating cell proliferation, migration, and angiogenesis. In the diabetic mouse wound model, TWEAK promotes angiogenesis and wound healing by regulating the Fn14/EGFR signal. TWEAK treatment significantly increases the expression of EGFR and Fn14 in human umbilical vein endothelial cells (HUVECs), while using EGFR inhibitors or Fn14 silencing can significantly weaken the pro-angiogenic effect of TWEAK (44). The NF-*κ*B signaling pathway is a classic pathway that initiates inflammatory responses and plays a key role in skin wound healing (45). Successful wound healing in the skin depends on the interaction between epidermal keratinocytes and dermal fibroblasts, and these cells generate a transient response through the NF-*κ*B pathway to initiate the healing process. The binding of TWEAK to Fn14 triggers the activation of both classical and non-classical NF-*κ*B pathways, generating pro-inflammatory factors, regulating cell proliferation, apoptosis, and migration (46, 47). There is currently no direct evidence indicating the relationship between the TWEAK/Fn14 signaling and NF-*κ*B in chronic wounds. NF-*κ*B activates the innate immune response, cell proliferation and migration, regulates the expression of matrix metalloproteinases, the secretion and stability of cytokines and growth factors, and promotes wound healing (48). In diabetic wounds, NF-*κ*B is continuously activated, and some drugs can promote M2 macrophage polarization by inhibiting NF-*κ*B signaling and related inflammatory factor expression, thereby reducing inflammation and promoting wound healing. This to some extent reflects the dual role of this signaling (49, 50). Additionally, TGF-*β*1 activates the expression of Fn14 through the transcription factor Smad4. The activation of Fn14 further increases the extracellular matrix (ECM) synthesis of dermal fibroblasts, verifying the activation of the ECM production by the TWEAK/Fn14 signaling (32). Studies have confirmed that the presence of amniotic membrane (AM) through epidermal growth factor (EGF) signaling transduction fine-tunes the TGF-*β* signaling pathway and re-directs the stalled wound healing process, further verifying its role in chronic wounds (51). The TWEAK/Fn14 signaling can also regulate the function of hair follicle stem cells (HFSC) through the Wnt/*β*-catenin-CXCR4 pathway, thereby enhancing the proliferation, migration, and secretion functions of stem cells, which is helpful for re-epithelialization and wound repair (52, 53). Moreover, TWEAK may upregulate E-selectin and intercellular adhesion molecule-1 on the surface of endothelial cells through the p38 signaling pathway and JNK signaling pathway, inducing the secretion of chemokines such as IL-8 and MCP-1, and inducing angiogenesis (54, 55). Regarding the PI3K/AKT pathway, studies have confirmed that the activation of the PI3K-Akt-mTOR pathway can significantly increase the proliferation, migration, and skin wound healing of epithelial cells (56). The PI3K/AKT pathway can promote the migration and proliferation of fibroblasts (57). In addition, insulin, PI3K/Akt/ras-related C3 toxin substrate 1 (rac1) and peroxisome proliferator-activated receptor-*γ* (PPAR-*γ*) signaling pathways are involved. This helps in the transition of M1 to M2 macrophages, accelerating the resolution of inflammation. Currently, most combined studies on these two factors are focused on tumors, while the specific mechanism of their action in chronic wounds requires further research (58). Hippo/YAP, as a core regulatory gene for macrophage function in diabetic foot ulcers (DFUs), induces M2 polarization and alleviates endothelial dysfunction, thereby promoting angiogenesis and tissue repair (40). TWEAK/Fn14 can eliminate inflammation and recruit macrophages, suggesting that there may be a synergistic effect between them in promoting wound healing.

## 4 TWEAK/Fn14 and chronic wound healing

### 4.1 Role of TWEAK/Fn14 in the inflammatory phase

**Figure 3:**
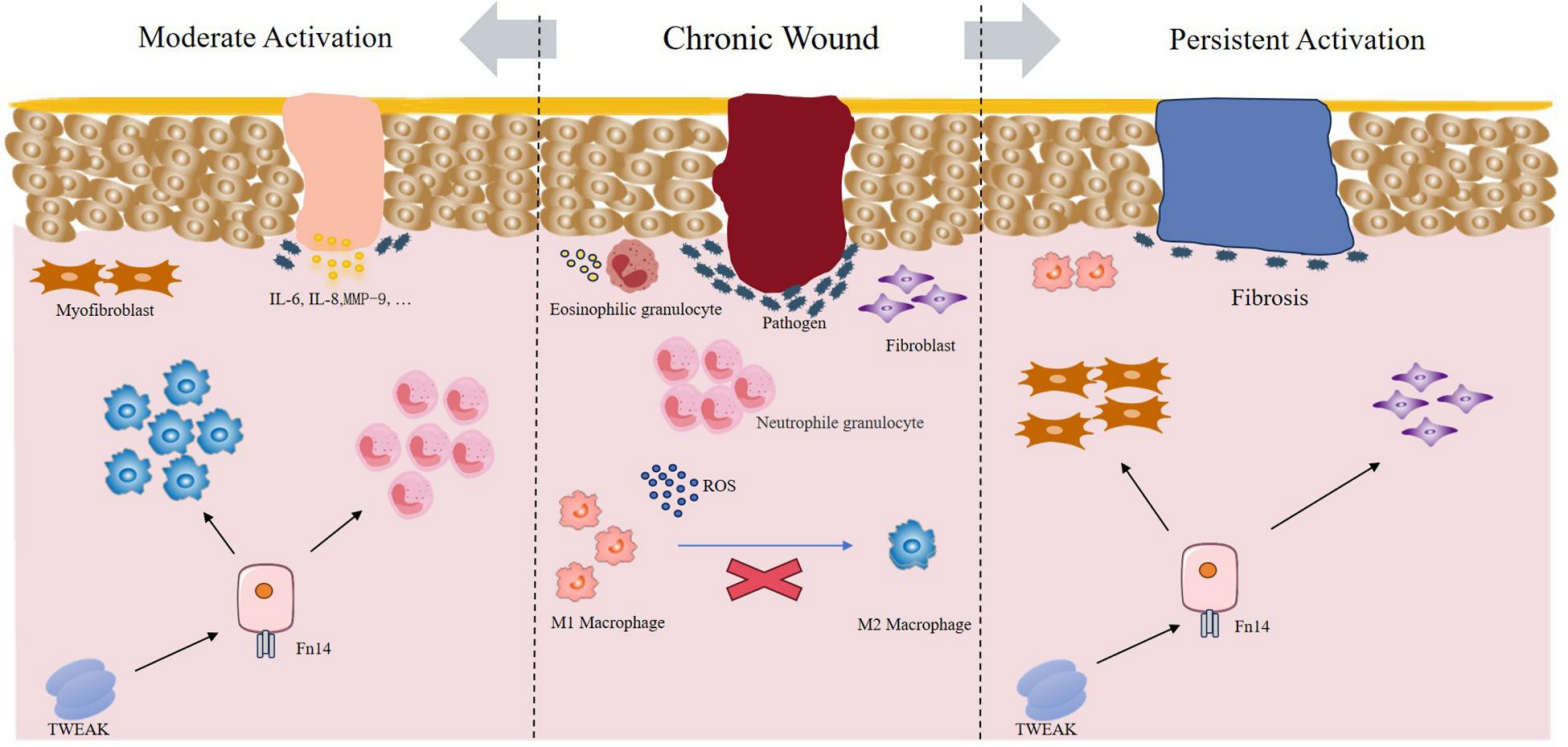
Mechanisms of TWEAK-Fn14 activation in chronic wound healing.

Chronic wound healing is the result of complex interactions among multiple cells, cytokines, and extracellular matrix. One characteristic of chronic wounds is the persistent inflammatory state. TWEAK and Fn14 are expressed in various inflammatory tissues, and studies have confirmed that fibroblasts, keratinocytes, endothelial cells, macrophages, and other cells can all express TWEAK and Fn14 (6, 59). During the inflammatory phase, the main role of TWEAK/Fn14 is to regulate inflammation and immune cell recruitment. TWEAK can activate the classical NF-*κ*B pathway by binding to Fn14, promoting the production of cytokines and chemokines and participating in the physiological responses of multiple cells (60). Under TWEAK stimulation, endothelial cells and fibroblasts migrate, inflammatory cells infiltrate, and the production of cytokines such as IL-6 and MCP-1 is induced. In keratinocytes, TWEAK promotes NF-*κ*B phosphorylation through Fn14 and upregulates multiple chemokines (MCP-1, RANTES, IP-10) and inflammatory cytokines (IL-6, IL-8), driving continuous recruitment of macrophages and neutrophils (30), clearing pathogens, and accelerating inflammatory progression. Although the TWEAK/Fn14 pathway has potential to promote productive tissue responses through coordinating controlled acute inflammation, angiogenesis, and fibrogenic responses after acute injury, as well as regulating parenchymal cell survival and growth and endogenous tissue progenitor cell responses, excessive or sustained activation of the TWEAK/Fn14 pathway in chronic injury diseases can amplify and dysregulate these events, leading to progressive harmful tissue injury and pathological tissue remodeling outcomes (59). Chronic fibrosis, as the ultimate pathological outcome of chronic inflammation, is promoted by the TWEAK/Fn14 axis through activation and proliferation of myofibroblasts, induction of myofibroblast secretion of tissue extracellular matrix, and regulation of pro-fibrotic mediators to further perpetuate and maintain the fibrotic process (61) (Figure 3). Therefore, during the inflammatory stage, TWEAK/Fn14 signaling has a dual role, with its intensity and duration determining its effects on chronic wounds.

### 4.2 Role of TWEAK/Fn14 in the proliferative phase

The proliferative stage involves large numbers of fibroblasts, keratinocytes, endothelial cells, and extracellular matrix (ECM) (62). Among these, myofibroblasts are key effector cells for wound contraction and matrix deposition, with their differentiation enhancing mechanical tension generation and collagen deposition, contributing to wound closure. During the proliferative phase, both TWEAK and Fn14 maintain high expression. TWEAK/Fn14 signaling significantly promotes fibroblast differentiation into myofibroblasts, with *α*-smooth muscle actin (*α*-SMA) and palladin expression both upregulated in a TWEAK dose- and time-dependent manner. This process relies on transcriptional regulation mediated by the NF-*κ*B, p38 MAPK, EGFR, and Smad4 pathways (63). Additionally, angiogenesis is also a characteristic of the proliferative phase. Zhu et al. (44) demonstrated in diabetic mouse models that TWEAK activates endothelial cell proliferation, migration, and lumen formation through Fn14, inducing expression of pro-angiogenic factors such as VEGF-A. Exogenous TWEAK administration significantly increased neovascular density and CD31-positive vessel numbers, promoting diabetic wound healing rates. Multiple studies have confirmed that TWEAK and Fn14 participate in angiogenesis during various repair processes (64–67). Although the proliferative phase is mainly characterized by regeneration and reconstruction, appropriate inflammation remains essential. TWEAK/Fn14 can induce expression of multiple cytokines and chemokines (such as IL- 6, CCL2, RANTES, IP-10), maintaining the inflammatory microenvironment (30).

### 4.3 Role of TWEAK/Fn14 in the remodeling phase

The remodeling phase is the final stage of wound healing, with ECM remodeling being the core process. Due to excessive MMP expression and prolonged activation in chronic wounds, excessive ECM degradation occurs, resulting in abnormal ECM component ratios and unstable wound matrix (68–70). Research on the mechanisms of TWEAK/Fn14 signaling in the remodeling phase of chronic wounds is limited. Currently, Liu et al. (63) demonstrated that local TWEAK application accelerates healing of experimental burn wounds in mice. The TWEAK/Fn14 signaling pathway promotes tissue tension recovery and accelerates wound healing by upregulating expression of hyaluronan synthase-1 (HAS-1), laminin *α*1, and collagen, while activating TWEAK/Fn14 can induce MMP-9 and ADAM17 expression, maintaining ECM synthesis and degradation balance. However, Shehata et al. (71) demonstrated that moderate TWEAK/Fn14 signaling contributes to normal healing during the remodeling phase, but sustained high expression can lead to pathological scar formation and chronic fibrosis, to some extent reflecting the dual role of TWEAK/Fn14 signaling.

## 5 Treatment of chronic wounds based on TWEAK/Fn14 signaling principles

In recent years, TWEAK/Fn14 has been recognized as associated with numerous pathological processes, such as muscle atrophy, cerebral ischemia, renal injury, atherosclerosis, myocardial infarction, and autoimmune diseases (including rheumatoid arthritis and inflammatory bowel disease) (72). Due to the dual nature of TWEAK/Fn14 signaling pathway effects, blocking or activating certain components of the TWEAK/Fn14 signaling pathway may become a new strategy for treating chronic wounds. Currently, in clinical and preclinical studies, TWEAK administration is divided into systemic treatment and local treatment (5), with exogenous TWEAK administration becoming the main therapeutic approach. Studies have demonstrated that topical TWEAK application can promote local inflammatory responses and cytokine production, synergistically accelerating healing of burn wounds and diabetic wounds (44, 63). Liu et al. demonstrated that TWEAK binds to and activates the surface cells of the lesion area, regulating the proliferation and differentiation of keratinocytes. The TWEAK/Fn14 signal enhances the migration of dermal microvascular endothelial cells (DMECs) and dermal fibroblasts *in vitro*. At the same time, the expression of TGF-*β*1, EGF, EGFR (phosphorylated), MMP-2 and MMP-9 in these cells is also promoted by TWEAK; knocking out Fn14 *in vitro* can eliminate this effect of TWEAK.

Although the role of TWEAK/Fn14 signaling in chronic wounds has been confirmed, due to its dual effects, related research remains very limited, and disease types are not comprehensive enough, with current studies focusing more on acute wounds. Antibodies or drugs developed based on this pathway are mostly used for anti-tumor and autoimmune diseases (81–83), and wound research needs further improvement. Additionally, the precise relationship between topical TWEAK dosage and healing efficacy, effects on wounds, and safety remain unknown, requiring further confirmation through more animal experiments and clinical trials.

## 6 Summary and prospects

The TWEAK/Fn14 signaling pathway plays a complex and important role in chronic wound healing, mainly by regulating inflammatory responses, angiogenesis, and multiple cell functions, participating in the wound repair process during inflammatory, proliferative, and remodeling stages. Currently, TWEAK/Fn14 research in chronic wound repair has achieved preliminary progress and may serve as a therapeutic target in the future. The main challenges facing translation of TWEAK/Fn14 signaling pathway-targeted therapeutic strategies into clinical applications are the complexity and interactions of signaling pathways in chronic wounds, the uncontrollability of TWEAK, limited efficacy of single-target interventions, and development of individualized treatment protocols. Although the role of TWEAK has been confirmed, related agents include monoclonal antibodies, fusion proteins, immunotoxins, and small molecule inhibitors (73–75), but most are concentrated on anti-tumor effects, with relatively limited wound repair-related applications. Furthermore, current research uses mouse models, which may not fully represent the complexity of human chronic wounds.

For the TWEAK/Fn14 signaling pathway, how to adjust the balance of this pathway has become a major challenge. When subjected to long-term stimulation, the common adverse reactions are its excessive promotion of inflammation and pathological fibrosis. To solve this problem, current research focuses on the following aspects: 1. TWEAK/Fn14 neutralizing antibodies or specific inhibitors. In multiple animal models, TWEAK blocking antibodies have shown significant therapeutic effects. These studies indicate that TWEAK neutralizing antibodies have therapeutic potential in reducing inflammation and preventing tissue damage (76–79), suggesting their possibility in the treatment of chronic wounds. 2. Reducing or knocking out the Fn14 molecule. In experimental mouse models, Fn14-deficient mice showed reduced inflammation and slower wound healing (54). Additionally, its application in chronic wounds still requires further research. 3. Utilizing TWEAK to construct an efficient and stable lentiviral transfection system or coupling with proteins. Liu et al. (80) demonstrated that LV-TWEAK-shRNA treatment could alleviate proteinuria and renal tissue pathological changes in patients with lupus nephritis, down-regulate the expression of TGF-*β*1, p-p38MAPK, and type I collagen, slow down the tissue fibrosis process in patients with lupus nephritis, and improve patient symptoms.

With deepening research, investigation of the interactions between the TWEAK/Fn14 pathway and the wound microenvironment, as well as synergistic effects with other signaling pathways, combined with novel multi-target combination strategies, will enable development of more effective chronic wound treatment protocols, becoming a research hotspot. Exploring new drug delivery systems that can precisely target the TWEAK/Fn14 pathway without inducing adverse reactions will become a research hotspot. At the same time, specific siRNAs or antisense oligonucleotides can be designed to down-regulate the expression of the Fn14 receptor or TWEAK ligand, thereby inhibiting the excessive activation of this signaling pathway in the local wound area. However, it should be noted that gene therapy still faces safety issues and efficacy challenges, and the delivery systems and treatment plans need to be further optimized. Additionally, local TWEAK dosage and its relationship with chronic wound healing efficacy and safety face challenges. Future research may investigate dosage-time relationships to formulate optimal protocols.
